# Fungal pathogens causing postharvest fruit rot of wolfberry and inhibitory effect of 2,3-butanedione

**DOI:** 10.3389/fmicb.2022.1068144

**Published:** 2023-01-10

**Authors:** Lijun Ling, Hong Luo, Yunhua Zhao, Caiyun Yang, Wenting Cheng, Mingmei Pang

**Affiliations:** ^1^College of Life Science, Northwest Normal University, Lanzhou, China; ^2^Bioactive Products Engineering Research Center for Gansu Distinctive Plants, Northwest Normal University, Lanzhou, China; ^3^New Rural Development Research Institute, Northwest Normal University, Lanzhou, China

**Keywords:** wolfberry, fungal pathogen, 2.3-butanedione, antifungal activity, postharvest fruit rot

## Abstract

Fungal pathogen contamination is one of the most important factors affecting the postharvest quality and shelf life of wolfberry fruits. Therefore, the prevention and control of fungal pathogens that cause fruit rot has become particularly important. Volatile antifungal agents of biological origin have broad application prospects. They may be safer and more efficient than traditional physical and chemical methods. Four pathogenic fungi were isolated and purified from rotting wolfberry. These pathogenic fungi were determined to be *Mucor circinelloides* LB1, *Fusarium arcuatisporum* LB5, *Alternaria iridiaustralis* LB7, and *Colletotrichum fioriniae* LB8. In vitro fumigation experiments showed that 2,3-butanedione can effectively inhibit the mycelial growth, spore germination, and sporulation ability of pathogenic fungi. The scanning electron microscope (SEM) showed morphological changes in hyphae. Propidium iodide (PI) Staining and leakage of 260 and 280 nm-absorbing increased, suggesting damage to cell membranes. Furthermore, 2,3-butanedione was found to significantly improve fruit firmness, soluble solid, total phenol, flavonoid, and soluble sugar content, as well as higher SOD enzyme activity and lower PPO and POD enzyme activity in the treated fruit, indicating that 2,3-butanedione can effectively reduce the adverse effects of pathogenic fungi in wolfberry. Based on these results, we conclude that 2,3-butanedione is effective against infection by pathogenic fungi in post-harvest wolfberry. 2,3-butanedione should be considered a viable substitute for conventional fungicides that are currently used to control rot in wolfberry.

**Figure:**
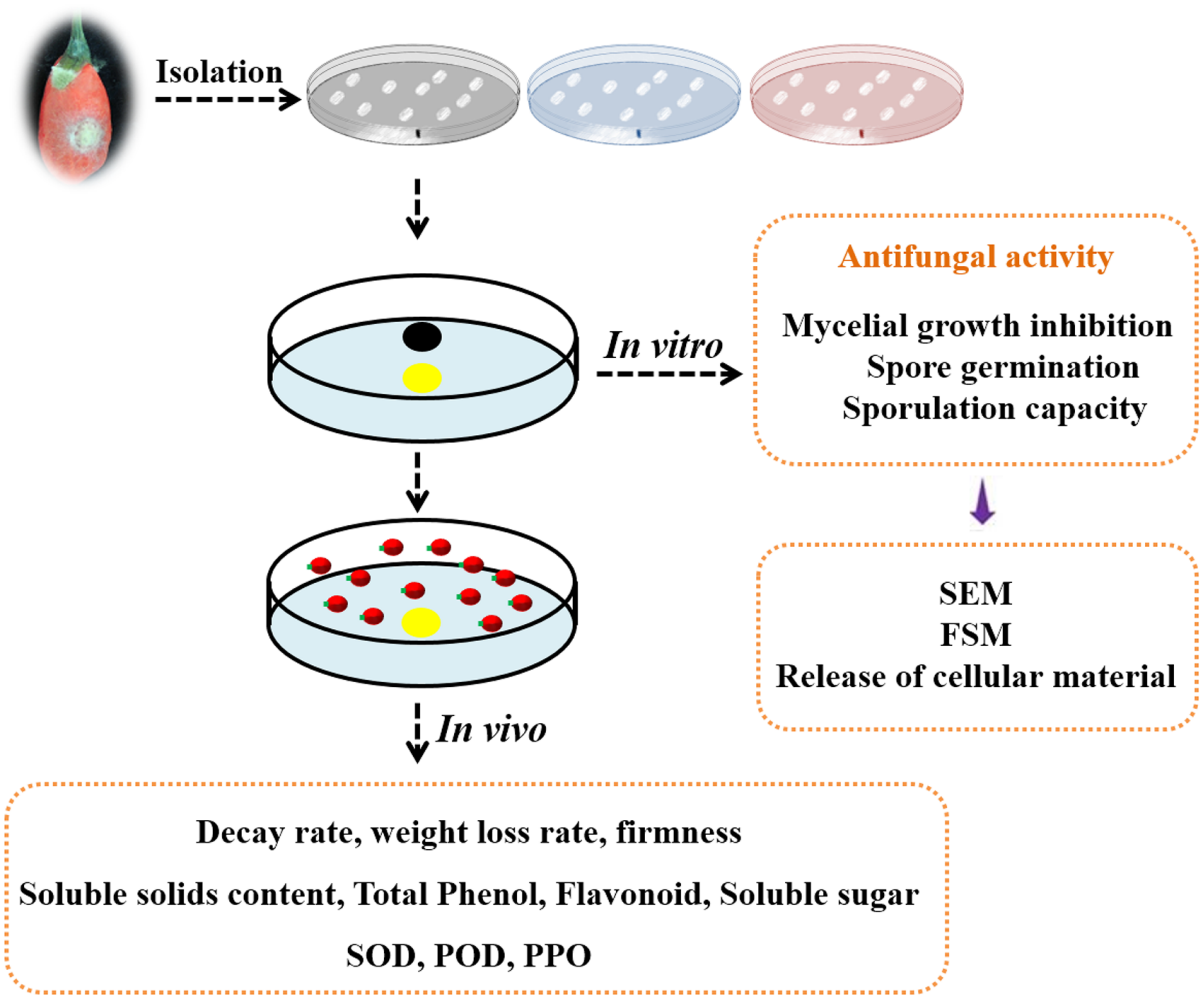
GRAPHICAL ABSTRACT

## 1. Introduction

Wolfberry (*Lycium barbarum* L.), often known as Goji Berry, is one of the most important plants belonging to the Solanaceae family (Liu et al., 2017a). For almost 2000 years, it has been an important Chinese traditional medicine, and it is widely grown in north-western China (Sun et al., 2008). Wolfberry is often used as a nourishing traditional Chinese medicine that has the function of nourishing the liver and kidney, tonifying blood, and relieving cough (Wang et al., 2014). At the same time, wolfberry can also be consumed as a functional food. Wolfberry fruit contains wolfberry polysaccharides, carotenoids, betaine, vitamins and other substances. Wolfberry polysaccharides have the ability of anti-oxidation to provide neuroprotection to the eyes (Chan et al., 2019). Because of its dual use as medicine and functional food, the development of the wolfberry industry has been promoted in recent years.

Postharvest loss refers to the decay and deterioration of agricultural products caused by pathogen infection, respiration and senescence in the process of storage, treatment and transportation after harvest (Zhang et al., 2019). Postharvest decay of harvested fruits and vegetables will cause food safety problems and considerable economic losses (Ambler et al., 2017). The postharvest infection of Chinese wolfberry is mainly caused by fungi. Several fungi have been reported to be associated with postharvest rot of wolfberry including *Alternaria*, *Aspergillus niger*, *Penicillium*, *Trichoderma*, *Aspergillus flavus* (Lan et al., 2014), *Cladosporium* and *Fusarium* (Xiao et al., 2018). However, there are still few reports on the main pathogenic fungi of wolfberry, and more research on it is necessary.

Keeping fruit fresh and disease-free throughout harvest and processing has long been a challenge. Currently, many strategies have been used to manage postharvest diseases of the wolfberry, such as low-temperature refrigeration, modified atmosphere refrigeration, physical treatments and chemical control (using synthetic fungicides), etc. (Yang et al., 2016). However, the most common and effective strategy for controlling postharvest diseases is the application of chemical agents in large quantities (Mari et al., 2016). Considering the negative side effects of fungicides, it is necessary to find a method that can replace fungicides to control postharvest pathogens of wolfberry fruit (Nunes, 2012).

In recent years, biological control has received extensive attention due to its safety, efficiency and environmental protection (Brilli et al., 2019). Numerous studies have shown that some antagonistic microorganisms exhibit strong inhibitory activity against postharvest pathogens in fruits and vegetables (Lemos Junior et al., 2020; Moore et al., 2021). Among them, some microorganisms capable of producing volatile organic compounds (VOCs) have great application prospects. Wonglom et al. (2020) report that VOCs released by *Trichoderma asperellum* T1 mediate antifungal activity and promote growth in lettuce. Zhong et al. (2021) reported that *Pseudomonas fluorescens* ZX produced VOCs against gray mold, effectively reducing the disease of grapes. Volatile organic compounds are not easy to remain, safe and efficient, which has a natural advantage in postharvest disease control (Wallace et al., 2017).

In the antifungal activity of VOCs, some constituent substances showed a strong effect. 2,3-butanedione is a naturally occurring and volatile α-diketone. Due to the aroma and flavor of butter, it is often used as a food additive and an important component of food flavors (Farsalinos et al., 2014). No adverse health effects have been reported at the level of 2,3-butanedione added to foods (Morgan et al., 2016). Our latest study found that endophytic *Bacillus subtilis* CL_2_ could significantly inhibit postharvest pathogens of wolfberry by producing VOCs, and the composition and function test of VOCs showed that 2,3-butanedione is the main active ingredient (Ling et al., 2021). 2,3-butanedione has always been used as an edible additive, and it is safe and harmless (Hallagan, 2017). But its powerful antifungal effect is not well understood. Therefore, it is very necessary to evaluate the postharvest disease control and preservation effect of this substance.

The goal of this research is to isolate and identify the pathogens that cause wolfberry fruit post-harvest rot, as well as to investigate the *in vitro* and *in vivo* antifungal activity of 2,3-butanedione against a fungal pathogen, in order to provide a theoretical foundation for the development of safe and healthy antisepsis technology for fresh wolfberry fruits.

## 2. Materials and methods

### 2.1. Postharvest rot-infected wolfberry

In August 2019, fresh wolfberry fruits were picked in Jingyuan County, Gansu Province, packed in foam boxes and brought back to the laboratory. After being stored in a 4°C refrigerator for 2 weeks, the rotten fruits were selected for the isolation of the pathogen.

2,3-butanedione was purchased from Niuniu Biochemical Co., Ltd. (Lanzhou, China). 2,3-butanedione (C_4_H_6_O_2_), C431038, Source: Macklin, molecular weight: 86.09, density: 0.985 g/ml, purity: ≥99.0%.

### 2.2. Pathogens isolation

The fruits of wolfberry with obvious rotting symptoms were disinfected with 75% ethanol for 1 min, and then rinsed with sterile distilled water (SDW) for 3 times; disinfected with 1% sodium sulfite for 5 min and rinsed with SDW 3 times. In an ultra-clean workbench, the rotting tissues were isolated with aseptic tweezers and transferred to Luria-Bertani medium (LB), yeast extract peptone glucose medium (YPD), and potato glucose agar (PDA) (Li et al., 2017). Colonies were transferred to media after 1 and 3 days of cultivation, and the process was repeated until a pure culture of strains was achieved.

### 2.3. Pathogenicity testing

The pathogenicity of the isolated strains was tested according to Koch’s rule. Sterilize the fresh wolfberry fruits with 75% ethanol for 1 min in a sterile environment, and rinse them with SDW 3 times. A sterile inoculating needle is used to pierce the surface of the fruit and inoculate the spore suspension of the isolated strain. Observe the pathogenicity of the isolated strains after incubating at 26°C for 3 d. The strains were inoculated on fresh and healthy wolfberry fruits again, and these pathogenic strains were isolated from the diseased wolfberry fruit tissues after inoculation. After purification and culture of these isolated strains, these pathogenic strains with the same morphological characteristics as the previously isolated strains were obtained, and the strain was identified as the pathogen of wolfberry fruits (María et al., 2018).

### 2.4. Pathogen identification

The DNA of pathogenic fungi was extracted by CTAB method (Ling et al., 2019). Use fungal ITS1 and ITS4 sequence primers for PCR amplification of strain DNA sequence (Cenobio-Galindo et al., 2019). The cycle parameters were as follows: an initial denaturation step at 95°C for 4 min, followed by 35 cycles of a denaturation step at 94°C for 30 s, an annealing step at 50°C for 60 s and an elongation step at 72°C for 80 s, followed by a final elongation step of 72°C for 8 min. PCR products were sent to Tianqi Gene Biotechnology Co., Ltd. for sequencing. Homologous sequences were analyzed against GenBank sequences using the NCBI nucleotide BLAST tool.^1^ Finally, these sequences were used to construct a phylogenetic tree using mega-7.0, and the obtained strains were analyzed and identified.

### 2.5. Minimum inhibitory concentration and minimum fungicidal concentration

For the minimum inhibitory concentration (MIC) and minimum fungicidal concentration (MFC) experiment, this was determined by the method of Zhou et al. (2017) with minor modifications. The PDA plates were inoculated in the centre with a 6 mm diameter disc of pathogen and then 0, 0.5, 0.75, 1, 1.25, 1.5, 1.75, 2, 2.5, 3, 3.5, 4, 4.5, 5 μl of the corresponding 2,3-butanedione were poured into the sterile filter paper on the lid of the Petri dish, followed by sealing with parafilm to prevent the loss of 2,3-butanedione. The Petri dishes were incubated at 28°C for 5 days, and after 48 h, the lowest 2,3-butanedione concentration that totally inhibited fungal growth was measured as the MIC, and after 96 h, the lowest concentration that entirely prevented fungal growth was calculated as the MFC.

### 2.6. Antifungal activity of 2,3-butanedione on spore germination and sporulation capacity

2,3-butanedione inhibition of fungal pathogens spore germination was measured using the method of Zhong et al. (2021) with minor modifications. PDA plates were coated with a pathogenic fungal spore suspension (1 × 10^4^ spores/ml). Add 2,3-butanedione (0, 1/4 MIC, 1/2 MIC, MIC) on sterile filter paper in the middle of the plate covering. The plates were sealed and incubated for 12 h at 28 degrees Celsius. A microscope was used to examine the spores. The proportion of sprouted spores out of the total number of examined spores was used to calculate germination rates.

Previous approaches were used to determine the sporulation capacity (Wang et al., 2020). A PDA plate was inoculated with a 6-mm-diameter plug of pathogenic fungus, then 2,3-butanedione was added on sterile filter paper in the middle of the plate covering. A 6 mm fungal plug was sliced and immersed in SDW after 5 days of incubation. A hemocytometer was used to count the spores on the plug.

### 2.7. Exploration of the influences of 2,3-butanedione on the mycelial morphology of pathogenic fungi by SSE of SEM technique

Fungal morphology was assayed by SEM, using our previously described method (Ling et al., 2021). At MIC, fungi that had been treated with 2,3-butanedione were cultivated and obtained. The fungal hypha was placed in a glass vial with a rubber stopper containing 2.5% glutaraldehyde fixation solution for 24 h at 4°C. The fixed hyphae were then washed with phosphate-buffered saline (PBS) three times for 15 min each time and dehydrated in an ethanol series (30, 50, 70, 85, and 90% and twice with 100% concentration) for 15 min at each stage. After natural drying, the mycelium block was pasted on a conductive adhesive, gilded, and then detected by SEM.

### 2.8. Plasma membrane integrity in treated and untreated

For plasma membrane integrity, the method of Tian et al. (2012) was applied with Propidium iodide (PI) staining. After the LB1 mycelia incubated in PDB were collected and washed, the mycelia were resuspended in sterile saline solution containing 5% Tween-20. Place sterile filter paper in the center of the cap, add 2,3-butanedione, and fumigate for 2 d. The mycelia were incubated with PI at a concentration of 100 μl/ml after incubation at 37°C for 30 min in the dark. The fungi were collected, and then washed three times with phosphate-buffered saline. Observe the samples with a fluorescence microscope.

### 2.9. Release of cellular material

The release of cellular material was tested with a slightly modified method by Kong et al. (2019). The corresponding fungal mycelia were collected and centrifuged from the PDB medium. Then, mycelia were washed twice with SDW and finally fumigated with different concentrations of 2,3-butanedione. Samples were collected and centrifuged with 8,000 g for 5 min. Measure the absorbance of the supernatant at 260 nm and 280 nm using a spectrophotometer. All tests were performed in triplicate.

### 2.10. Decay rate, weight loss rate and firmness determination

Taking 30 wolfberry fruits in a sterile culture dish and inoculating pathogenic fungus LB1 on wolfberry fruits (without any treatment as a control). The fruit was fumigated with 2,3-butanedione in a closed environment. Any fruit with visible mycelium growing on the surface or rotted area exceeding 12.5% of the surface area was considered as rotted fruit. The decay weight and mass loss of wolfberry fruits were calculated by the following formula.

The firmness of wolfberry fruits treated with 2,3-butanedione for different times were measured using a GY-3 fruit firmness tester (Wei et al., 2018). Determination when taking two wolfberry fruits overlap together, with fruit firmness tester vertical squeeze wolfberry fruit, uniform force will probe into the wolfberry fruits, when the fruit firmness tester probe just through the two fruits stop squeezing, record fruits firmness tester pointer pointed to the reading, repeat three times.


$$\mathrm{Decay rate}\;\left(\%\right)=\left(\mathrm{number of rotten fruits}/\mathrm{total fruits}\right)\times 100$$

$$\mathrm{Weight loss}\;\left(\%\right)=\left[\begin{array}{l} \left(\mathrm{initial weight}-\mathrm{final weight}\right)\\ /\mathrm{initial weight} \end{array}\right]\times 100$$

### 2.11. Soluble solids content and soluble sugar content determination

The effect of 2,3-butanedione treatment on the content of soluble solids in wolfberry fruits were determined by a handheld refractometer. 5 g of wolfberry fruits were weighed into a 2 ml centrifuge tube, ground with a grinding rod until homogenized, centrifuged at 2000 × g for 5 min and then 5 μl of wolfberry fruits homogenate was aspirated and added dropwise to the detection mirror of the handheld refractometer, and the scale was read to determine the amount of soluble solids in the sample solution, expressed as mass fraction (%), with three replicates for each group of treatments (Chen et al., 2015).

The content of soluble sugars in wolfberry fruits was determined by anthrone-sulfuric acid method. In a test tube, 0.5 g of wolfberry fruits were weighed and ground into a homogenate with SDW and boiled for 30 min, then filtered, the residue was recovered and boiled again for 10 min and repeated three times. Pour the filtered boiling extract into a 50 ml volumetric flask and fix the volume with SDW. Pipette 50 μl of the sample extract of wolfberry soluble sugar into a test tube, and then add 0.5 ml of anthranilone-ethyl acetate and 5.0 ml of concentrated sulfuric acid to the test tube in turn. After shaking thoroughly, the tubes were immediately removed from the boiling water bath for 1 min and cooled naturally to room temperature. The absorbance value was measured at the wavelength 630 nm and the soluble sugar content in the fruit of wolfberry fruits was expressed as *OD*_630_.

### 2.12. Total phenol and flavonoid determination

Weigh 1 g wolfberry fruits in a 10 ml centrifuge tube, add 1% hydrochloric acid-methanol solution under the condition of ice bath, grind and mix well. After placing in the dark at 4°C for 30 min, shake three times during the period and collect the filtrate was for later use. Then the filtrate was diluted 5 times with 1% hydrochloric acid-methanol solution, the absorbance at 325 nm was measured by an ultraviolet spectrophotometer, and *OD*_325_ was used to represent the flavonoids content in wolfberry fruits. The filtrate was diluted 10 times with 1% hydrochloric acid-methanol solution was taken to determine the absorbance at 280 nm, and *OD*_280_ was used to represent the total phenol content in wolfberry fruits (Habibi et al., 2019).

### 2.13. SOD, POD, and PPO determination

Weigh 5 g wolfberry fruits in a centrifuge tube, added with 5 ml of extraction buffer and then ground and homogenized under ice bath conditions. Centrifuge at 9614 g for 30 min at 4°C, collect supernatant, and store it at low temperature for later use.

Place each solution in the test tube as listed (Supplementary Table 1). Two test tubes were taken as control tubes. After uniform mixing, one control tube was placed in the dark, and the other test tubes were placed under a 4,000-lx fluorescent lamp for reaction for 15 min, and then immediately placed in the dark to terminate the reaction. Determine the absorbance at 560 nm with an unlit tube as the blank reference zero. Three replicates. SOD activity was calculated (Wu et al., 2020).

Add 3 ml of 25 mmol/l guaiacol solution and 0.5 ml of enzyme extract to the test tube, then 200 ml of 0.5 mol/l H_2_O_2_ solution to quickly initiate the reaction and start the timer. The initial value was recorded as the absorbance at 470 nm for 15 s, followed by 1 min of recording and 6 continuous measurements. To calculate the POD activity, the process was done three times.

Place 4.0 ml of 50 mmol/l acetic acid–sodium acetate buffer, pH 5.5, and 1.0 ml of 50 mmol/l catechol in the test tube, and add 100 μl of the enzyme extract, beginning immediately. Absorbance at 420 nm for 15 s is the initial value and is recorded at regular intervals for at least six times. And repeat for three times to calculate that PPO activity (Fan et al., 2019).

### 2.14. Statistical analyses

The SPSS 20.0 software was used to conduct statistical analyses. Duncan’s test was applied to compare the mean values. All data were expressed as the mean ± SE by measuring three independent replicates.

## 3. Results and discussion

### 3.1. Isolation of pathogens and pathogenicity testing

Wolfberry is a traditional Chinese medicinal herb recognized as the latest “super-fruit” in the world. Due to the tender peel and high-water content, the fresh wolfberry fruits are easy to rot (Jatoi et al., 2017; Liu et al., 2017b). Most pathogenic microbes that cause post-harvest deterioration of wolfberry are fungi. Some pathogenic fungi also yield mycotoxins that convey a risk to human health (Liu et al., 2018). In order to better preserve and store wolfberry after harvest, more pathogenic fungi of wolfberry fruit were isolated, and a safe 2,3-butanedione was used for postharvest control of wolfberry in vapor phase.

**Figure 1:**
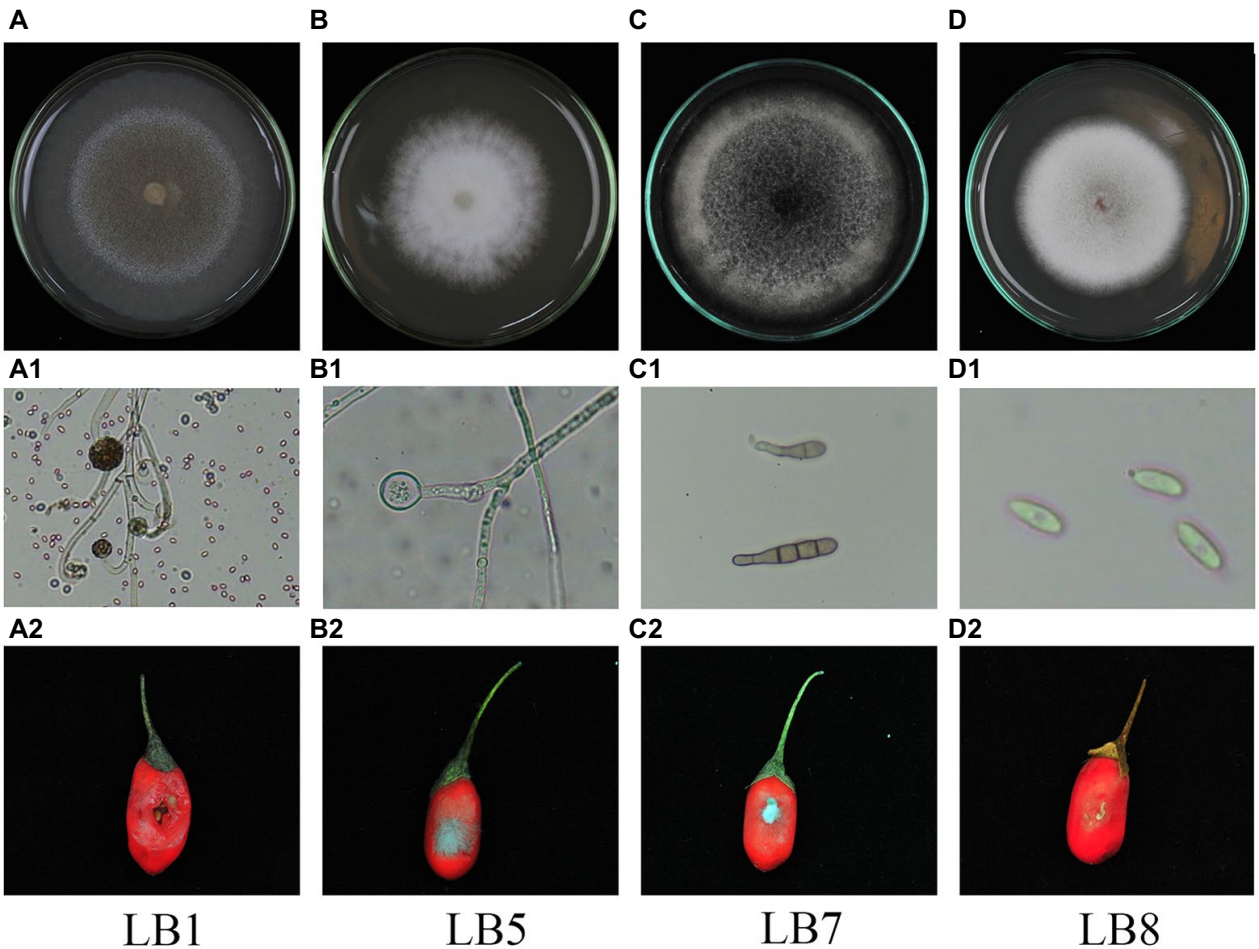
Morphological characteristics and pathogenicity detection of pathogenic strains of wolfberry after harvest (**A–D**: surface morphology of pathogenic strains, **A1–D1**: hyphae and spore morphology of pathogenic strains in microscopic morphology, **A2–D2**: observation of pathogenicity of pathogenic strains on fruits).

After morphological observation and pathogenicity detection, 4 strains of postharvest pathogenic fungi were isolated. They were numbered LB1, LB5, LB7 and LB8. The morphology of 4 pathogenic fungi isolated from wolfberry fruits was shown in Figure 1. The pathogenic fungal LB1 mycelium grows quickly, and the colony color was light yellow (Figure 1A), after about 3 d of growth, a black floating layer appeared on the surface of the mycelium and the colonies could be removed directly from the PDA medium by forceps. A lot of oval spores with cystspores were observed under optical microscopes, and mycelia were seen separable (Figure 1A1). The colony of LB5 looked like white cotton bats (Figure 1B). The hyphae were long and separated, and the spores were scattered (Figure 1B1). Colonies of LB7 on PDA medium were black or dark green and fluffy (Figure 1C), under the light microscope, oval-shaped dark brown conidia could be seen, and many spores were connected (Figure 1C1). The mycelia of the pathogen LB8 were short and felt-like, and the middle color was pink (Figure 1D), as the growth time increased, the surface of the colony became rough, and black particles appeared. Oval spores of LB8 could be seen under a light microscope (Figure 1D1).

After inoculating pathogens for some time (LB1 treatment for 2 d, LB5, LB7 and LB8 treatment for 3 d), Fruits of wolfberry inoculated with pathogens showed different degrees of decay, as shown in Figure 1. As shown in Figure 1A2, the fruit of wolfberry began to rot 1 d after inoculation with pathogen LB1, and on the next day, nearly half of the surface of the fruit was seriously rotted, and a large amount of juice began to precipitate out of the fruit, but there was no mycelium visible to the naked eye. As shown in Figure 1B2, 3 d after inoculation with pathogen LB5, the surface of wolfberry fruit was covered by a large number of hyphae, and the fruit tissue around the hyphae began to soften. In figure (Figure 1C2) and (Figure 1D2), mycelia appeared on the fruit surface of wolfberry 3 d after inoculation with pathogens LB7 and LB8, and the fruit tissue around the pathogen became black and began to soften.

### 3.2. Identification of the postharvest fungal pathogens

**Figure 2:**
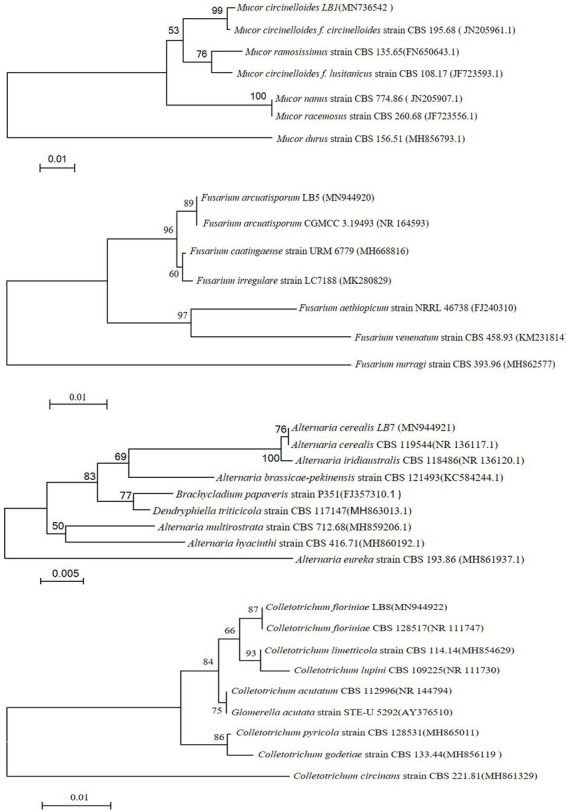
Phylogenetic tree of pathogenic fungi in wolfberry postharvest.

In order to further characterize the isolates, phylogenetic trees were constructed from species using closely related relatives (Figure 2). The isolated strains show high genetic homology with the known strain sequences in the phylogenetic tree. According to the identification studies, of the 4 pathogenic fungi isolates, LB1 was identified as *Mucor circinelloides,* LB5 was identified as *Fusarium arcuatisporum,* LB7 was identified as *Alternaria iridiaustralis,* and LB8 was identified as *Colletotrichum fioriniae.*

The ITS nucleotide sequences of the isolates have the following accession numbers and GenBank database accession codes: LB1 and MN736542, LB5 and MN944920, LB7 and MN944921, and LB8 and MN944922.

Chinese wolfberry pathogenic fungi were isolated and identified in this study. *M. circinelloides*, *F. arcuatisporum*, *A. iridiaustralis*, and *C. fioriniae* were identified as the main pathogens causing postharvest wolfberry rot. Previous research has shown that *P*., *Alternaria*, *A. niger*, *Trichoderma* and *Aspergillus* were the main post-harvest pathogenic fungi in Chinese wolfberry (Lan et al., 2014). *Fusarium*, *Alternaria*, *Penicillium*, *Gibberella*, *Fusarium oxysporum*, *Penicillium oxalicum*, *Cladosporium*, *Alternaria pallidus* and *Alternaria alternata* were isolated from post-harvest wolfberry (Liu et al., 2018; Xiao et al., 2018). Compared with previous studies, this was the first report of isolation and identification of *Mucor circinelloides* and *Colletotrichum fioriniae* from postharvest wolfberry. It is worth noting that the identified pathogenic fungi LB1 showed a strong fungal infection to cause the decay of the wolfberry fruit harvested. Therefore, this study chose to explore the inhibition of strain LB1 *in vitro* and *in vivo*.

### 3.3. Inhibition of 2, 3-butanedione on pathogenic fungi

Table 1 shows the results of 2,3-butanedione in the vapor phase that showed inhibitory efficacy against pathogenic fungus. For the MIC and MFC experiments, 2,3-butanedione was observed to significantly inhibit the growth of strains LB5, LB7 and LB8, while strain LB1 required more 2,3-butanedione to achieve the same inhibitory effect. After 48 h of incubation at 28°C, the lowest concentration of 2,3-butanedione conferring complete inhibition of LB1 was 4.5 μl per plate (MIC). After 96 h of incubation, 2,3-butanedione at 5 μl per plate completely controlled the growth of LB1, yielding a minimum fungicidal concentration (MFC) of 5 μl per plate.

**Table 1:** Minimum inhibitory concentration and minimum fungicidal concentration of 2,3-butanedione against pathogenic fungus.

Concentration (μl per plate)	Mycelial growth inhibition (%)*							
	LB-1		LB-5		LB-7		LB-8	
	48 h	96 h	48 h	96 h	48 h	96 h	48 h	96 h
0.5	0^a^	0^a^	33.33 ± 0.18^a^	27.78 ± 0.16^a^	28.61 ± 0.19^a^	20.41 ± 0.25^a^	33.41 ± 0.17^a^	36.67 ± 0.2^a^
0.75	9.25 ± 0.02^b^	0^a^	60.00 ± 0.24^b^	48.27 ± 0.16^b^	42.86 ± 0.18^b^	32.65 ± 0.12^b^	70.62 ± 0.26^b^	56.67 ± 0.18^b^
1	11.11 ± 0.19^c^	2.33 ± 0.19^b^	93.33 ± 0.13^c^	86.62 ± 0.21^c^	50.22 ± 0.26^c^	40.82 ± 0.13^c^	90.78 ± 0.2^c^	83.53 ± 0.18^c^
1.25	24.07 ± 0.13^d^	10.67 ± 0.19^c^	100^d^	97.22 ± 0.18^d^	64.38 ± 0.25^d^	46.67 ± 0.18^d^	100^e^	96.54 ± 0.19^e^
1.5	37.04 ± 0.24^e^	24.37 ± 0.14^d^	100^d^	100^e^	85.71 ± 0.16^e^	66.67 ± 0.24^e^	100^e^	100^f^
1.75	40.74 ± 0.21^f^	31.59 ± 0.21^e^	100^d^	100^e^	100^f^	71.37 ± 0.20^f^	100^e^	100^f^
2	48.15 ± 0.23^g^	39.69 ± 0.18^f^	100^d^	100^e^	100^f^	86.57 ± 0.18^g^	100^e^	100^f^
2.5	62.96 ± 0.15^h^	46.22 ± 0.18^g^	100^d^	100^e^	100^f^	98.34 ± 0.19^h^	100^e^	100^f^
3	70.37 ± 0.14^i^	61.34 ± 0.19^h^	100^d^	100^e^	100^f^	100^i^	100^e^	100^f^
3.5	88.89 ± 0.23^j^	74.79 ± 0.23^i^	100^d^	100^e^	100^f^	100^i^	100^e^	100^f^
4	98.15 ± 0.23^k^	88.46 ± 0.18^j^	100^d^	100^e^	100^f^	100^i^	100^e^	100^f^
4.5	100^l^	98.16 ± 0.20^k^	100^d^	100^e^	100^f^	100^i^	100^e^	100^f^
5	100^l^	100^l^	100^d^	100^e^	100^f^	100^i^	100^e^	100^f^

Data are expressed as means of three replicates ± SD. ^a − i^ Values within a column followed by different letters are significantly different (*p* < 0.05).

### 3.4. Effects of 2, 3-butanedione on spore germination and sporulation capacity of pathogenic fungi

As shown in Tables 2, 3, the effect of 2,3-butanedione on spore germination and sporulation capacity of pathogenic fungi was determined. Treatment with 2,3-butanedione at all concentrations (0, 1/4 MIC, 1/2 MIC, MIC) tested resulted in significantly lower spore germination rates and sporulation compared to untreated. The inhibitory effect was positively correlated with the concentration of 2,3-butanedione. When the concentration reached MIC value, spore germination and sporulation of LB1 were 14.21% and 39.35 spores/cm^2^, respectively, and both values were significantly lower (*p* < 0.05) than those of control (96.8% and 270.83 spores/cm^2^, respectively).

**Table 2:** Effects of 2,3-butanedione on the spore germination.

Concentration of 2,3-butanedione (μl per plate)	Spore germination (%) incubated for 12 h			
	LB-1	LB-5	LB-7	LB-8
0	96.80 ± 2.88^a^	91.71 ± 3.70^a^	97.50 ± 2.43^a^	94.11 ± 4.15^a^
1/4 MIC	82.40 ± 4.19^b^	40.80 ± 2.32^b^	73.40 ± 4.57^b^	81.75 ± 3.20^b^
1/2 MIC	48.80 ± 2.64^c^	10.93 ± 3.23^c^	21.33 ± 3.45^c^	35.33 ± 4.88^c^
MIC	14.21 ± 1.94^d^	0.00 ± 0.00^d^	6.45 ± 2.15^d^	4.80 ± 1.88^d^

Data are expressed as means of three replicates ± SD. ^a − d^ Values within a column followed by different letters are significantly different (*p* < 0.05).

**Table 3:** Effects of 2,3-butanedione on the sporulation capacity.

Concentration of 2,3-butanedione (μl per plate)	Sporulation capacity (×10^4^ spores/cm^2^) incubated for 4 d			
	LB-1	LB-5	LB-7	LB-8
0	270.83 ± 4.01^a^	7.87 ± 1.67^a^	55.44 ± 4.88^a^	100.92 ± 2.45^a^
1/4 MIC	167.02 ± 3.71^b^	3.42 ± 0.40^b^	32.41 ± 2.45^b^	50.75 ± 3.21^b^
1/2 MIC	111.46 ± 6.01^c^	2.13 ± 0.24^bc^	10.85 ± 0.42^c^	20.96 ± 1.26^c^
MIC	39.35 ± 2.32^d^	0.32 ± 0.12^c^	3.24 ± 1.23^c^	2.32 ± 0.46^d^

Data are expressed as means of three replicates ± SD. ^a − d^ Values within a column followed by different letters are significantly different (*p* < 0.05).

### 3.5. Effects of 2, 3-butanedione on mycelial morphology of pathogenic fungi

**Figure 3:**
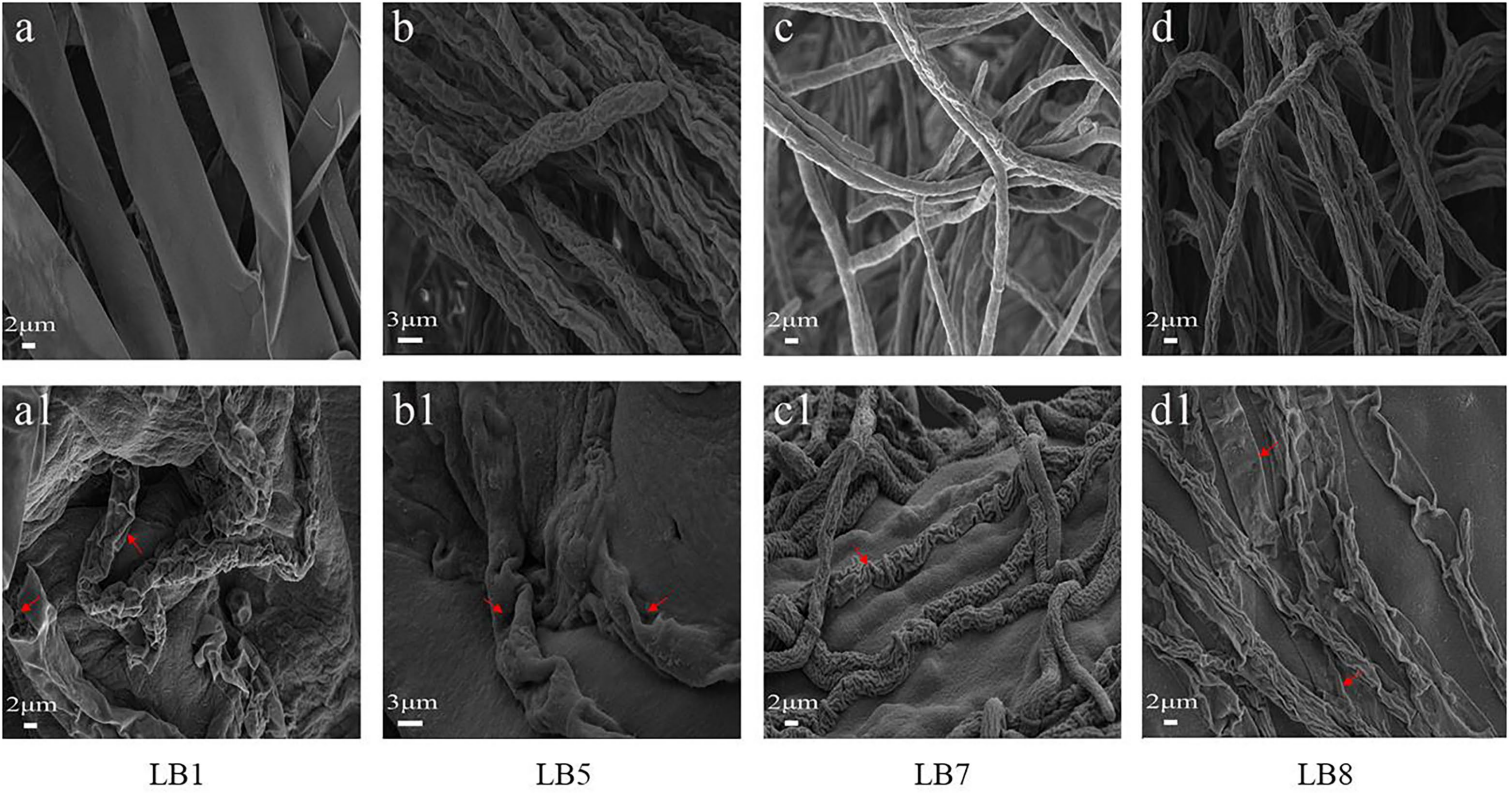
Scanning electron microscopy observation of mycelial morphology (**A–D**: control group, **A1–D1**: treatment group with 2,3-butanedione).

Figure 3 depicts the morphological changes seen in pathogenic fungi treated with 2,3-butanedione MIC values. The hyphal morphology of the four pathogenic fungi changed considerably when compared to the control fungi. In comparison to the control group, 2,3-butanedione forced the mycelium of pathogenic fungi LB1 to lose linearity, the surface of the mycelium to become frizzy, and the hyphae wall to become sabotaged (Figures 3A, A1); the mycelium of LB5 to swell and the surface of the hyphae to become sunken (Figures 3B, B1); The hyphae of LB7 were wrinkled and concomitantly sunken (Figures 3C, C1), The mycelia of the pathogenic fungus LB8 were severely distorted and appeared to be severely collapsed and squashed (Figures 3D, D1).

### 3.6. Effects of 2,3-butanedione on plasmalemma integrity of LB1

**Figure 4:**
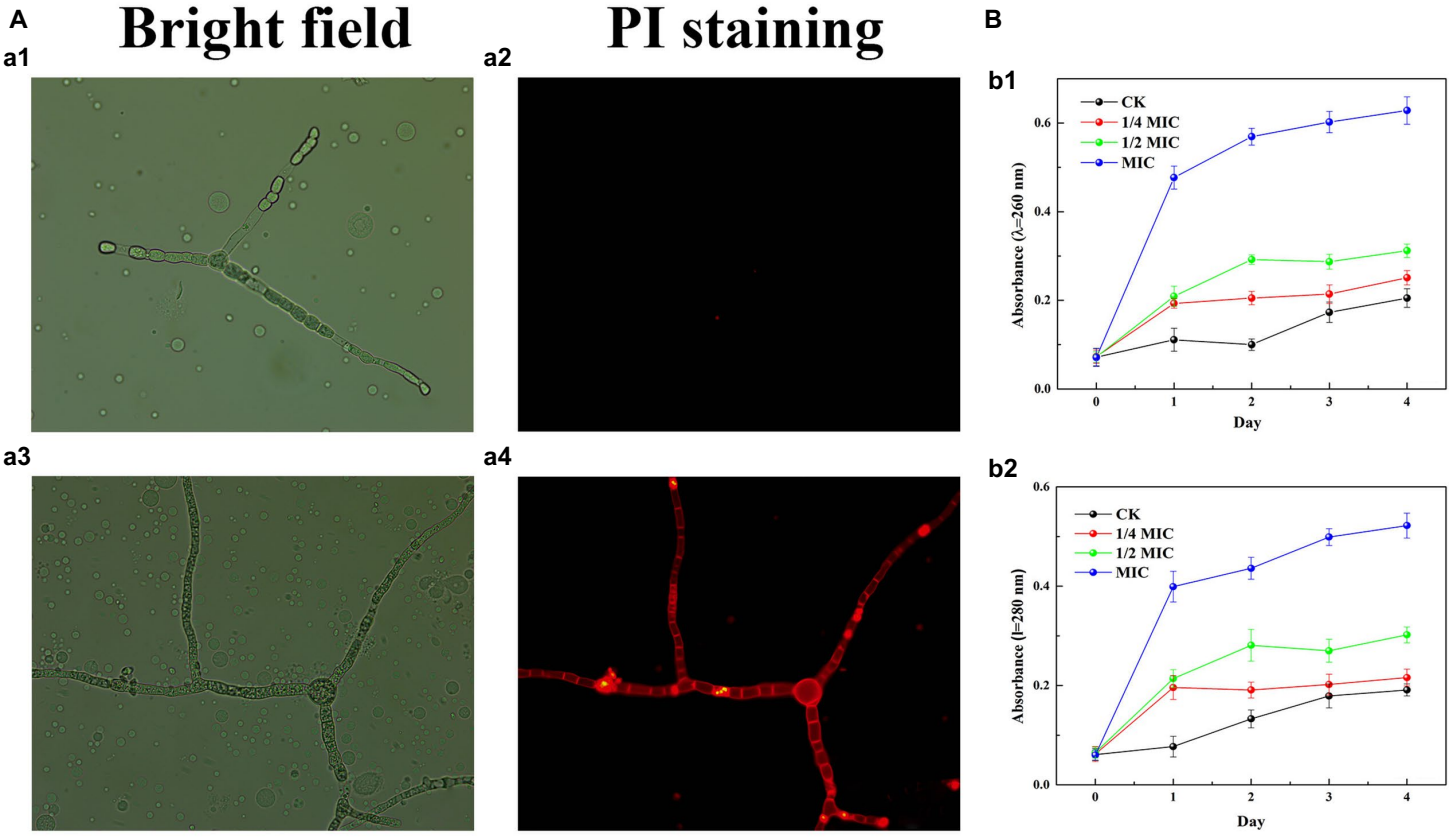
**(A)** Effects of 2,3-butanedione on plasma membrane integrity (**A1**, **A2**: control group, a3-a4: treatment group with 2,3-butanedione, First row: bright field. Second row: propidium iodide). **(B)** Determination of absorbance value at 260 nm **(B1)** and 280 nm **(B2)** by 2,3-butanedione treatment.

Propidium iodide (PI) staining was used to assess the integrity of the plasma membrane by 2.3-butanedione treatment. As shown in Figure 4A, the hyphae of the control group were solely a small portion of red fluorescence while the red fluorescence in the 2,3-butanedione treatment group was remarkably higher. The results indicated that 2,3-butanedione could compromise the integrity of the plasma membrane.

### 3.7. Release of cell constituents

The internal components of the cell are released when the cell membrane is ruptured by 2,3-butanedione (Figure 4B). In comparison to the control group, the *OD_260_* and *OD_280_* values rise considerably with treatment time and 2,3-butanedione concentration. This shows that strain LB1 has lost proteins and nucleic acids.

Chaouachi et al. (2021) reported that the *Enterobacter* strain TR1 with 3-methylbutan-1-ol as the main volatile compound has the strongest protective effect on the growth and infection of tomato *Botrytis cinerea*. Zhao et al. (2019) reported that 2, 4-di-tert-butylphenol can significantly inhibit the germination of fungal spores, and change the hyphal morphology and membrane permeability. Essential oils of plants have also been reported to maintain the same antifungal properties (Xu et al., 2021). 2, 3-Butanedione has a strong inhibitory effect on the pathogenic fungi of wolfberry. With the increase of the concentration, it can seriously hinder hyphal growth, spore pullulation and sporulation. SEM results showed that 2,3-butanedione could result the hyphae to shrink, depression, severely collapse and flatten. This result is consistent with other reports on the effects of fungistatic substances. The plasma membrane plays an important role in maintaining the normal life activities of cells. Fluorescence microscopy (FSM) showed that 2,3-butanedione can damage plasma membrane. In addition, the *OD_260_* and *OD_280_* absorbance values increased, indicating that the increase in cell membrane permeability resulted in the release of internal components. 2,3-Butanedione acts on the cell membrane of pathogenic fungi to produce an inhibitory effect. This is not only consistent with our results, but also consistent with other reports on the effects of antifungal substances (Cui et al., 2021; Li et al., 2021).

### 3.8. Decay rate, weight loss rate and firmness

**Figure 5:**
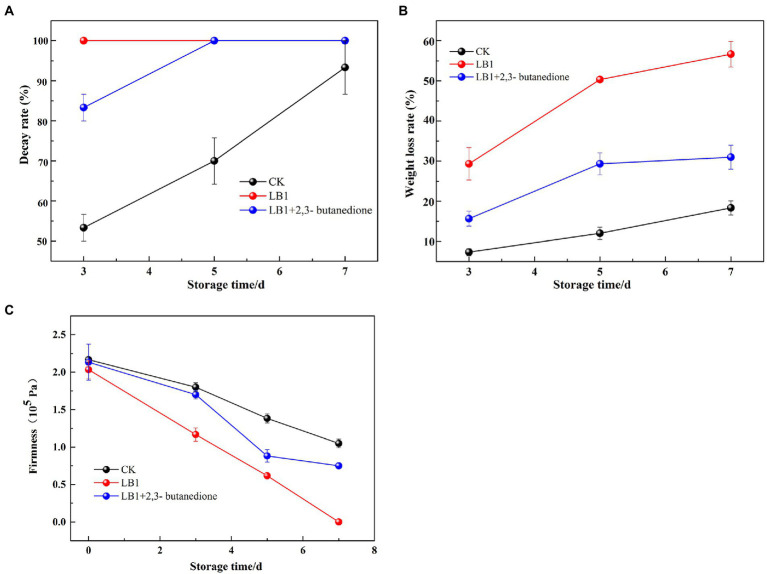
Determination of Decay weight, weight loss and firmness of wolfberry fruit after harvest (**A**: Decay weight, **B**: weight loss, **C**: firmness).

Decay rate as shown in Figure 5A, on the 3rd day of treatment, the wolfberry fruits in the group inoculated with the pathogenic fungus LB1 had completely rotted, while the 2,3-butanedione treated group had completely rotted on the 5th day.

The mass loss rate of the fruits induced by strain LB1 was 29.3% on the 3rd day of treatment, while the 2,3-butanedione treated groups was 15.7%. On the 7th day of treatment, the weight loss rate of 2,3-butanedione treated wolfberry fruit was 25.7% lower than that of the wolfberry fruit inoculated with strain LB1 (Figure 5B).

The firmness of wolfberry fruits in the group inoculated with pathogenic fungi LB1 decreased from 2.03 × 10^5^ Pa to 1.16 × 10^5^ Pa after 3 days of treatment, and the firmness was almost undetectable on the 7th day. In contrast, the firmness of the fruit in the 2,3-butanedione treated group was 0.75 × 10^5^ on the 7th day, which was significantly higher than that in the former (Figure 5C). Strain LB1 considerably enhanced the decay rate and weight loss rate, as well as reducing the hardness of wolfberry fruits, as seen in Figure 5. 2,3-butanedione slowed the deterioration of wolfberries induced by strain LB1, decreased the rate of water analysis, kept the firmness of the fruits, and extended the wolfberry’s storage time.

### 3.9. Soluble solids content, total phenol, flavonoid and soluble sugar content

**Figure 6:**
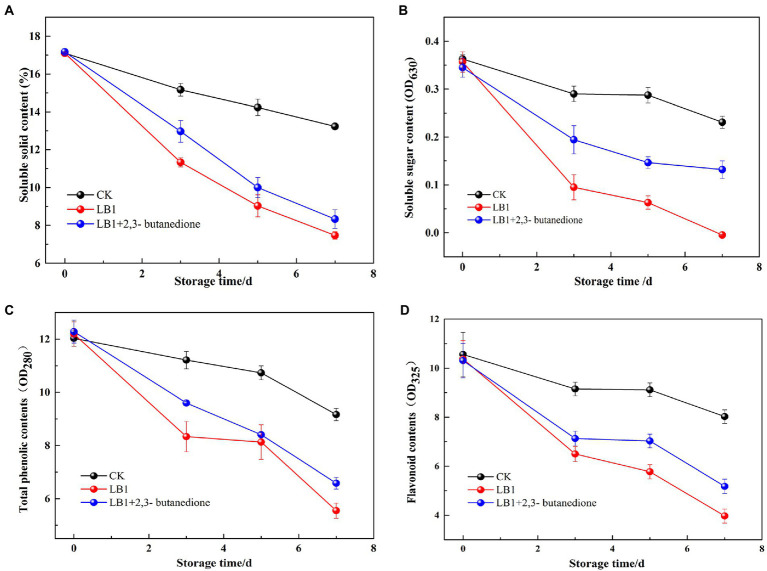
Determination of the soluble solids content, total phenols, flavonoids and soluble sugar content of wolfberry fruits after harvest (**A**: soluble solids content, **B**: total phenols, **C**: flavonoids, **D**: soluble sugar content).

As can be seen from Figure 6, the soluble solids content, soluble sugar content, total phenol and flavonoid in wolfberry fruits showed an overall decreasing trend during the storage process. The soluble solids content, soluble sugar content, total phenol and flavonoid in the 2,3-butanedione treated group were significantly higher than the pathogenic fungi LB1 treated group. However, they were all lower than the control group that had been left blank. This implies that 2,3-butanedione can reduce the rate of nutrient loss caused by the pathogenic LB1 strain.

The disintegration rate, mass loss rate, firmness and soluble solids content of wolfberry fruits directly expressed the quality of wolfberry fruits and affected the storage time of wolfberry fruits. Soluble sugars are the basic substance of the respiration of wolfberry fruits, while the secondary metabolites such as total phenol and flavonoid are closely related to the color development, ripening and aging, flavor formation and stress resistance of wolfberry (Liu et al., 2021). Wang et al. (2012) found 1-methylcyclopropene (1-MCP) could delay the decay time of fresh wolfberry fruits, but it was not a natural product and had low toxicity. Xiao et al. (2018) found that salicylic acid (SA) had an inhibitory effect on pathogenic fungi of wolfberry fruits and could reduce the occurrence of postharvest diseases. However, SA had no meaningful impact on the content of soluble solids in wolfberry fruits, and had a certain irritant to human skin. 2,3-butanedione can virtually stymie the growth and reproduction of pathogenic strain LB1 in wolfberry fruits, delay the utilization and consumption of nutrients in fruits and prolong the storage time of wolfberry fruits. 2,3-butanedione has a small molecular weight, is easy to volatilize, avoids the residue on the surface of the fruit, and is a commonly used food flavor ingredient. Therefore, it is expected to be used as a preservative and biocontrol agent for wolfberry fruit.

### 3.10. SOD, POD and PPO

**Figure 7:**
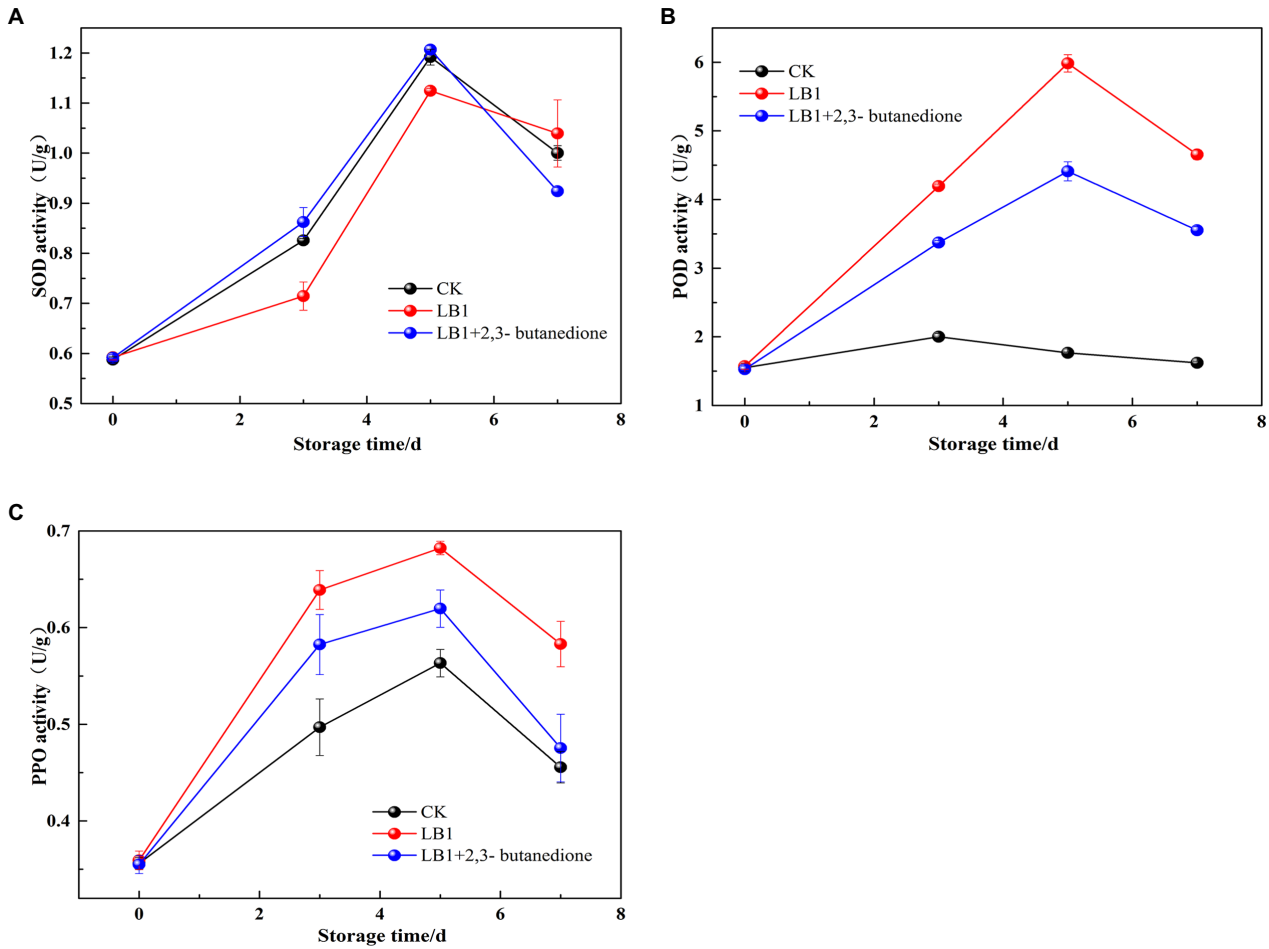
Determination of Peroxidase (POD), polyphenoloxidase enzymes (PPO) and superoxide dismutase (SOD) of postharvest wolfberry fruits (**A**: SOD, **B**: POD, **C**: PPO).

The SOD, PPO, and POD activity in wolfberry fruits increased and then decreased after storage, as seen in (Figure 7). The SOD activity of the 2, 3-butanedione treated postharvest fruits were higher in the first 5 days of storage than the control and inoculation strain LB1 treated groups, but decreased on the 7th day. At the beginning of storage, the 2,3-butanedione treatments retained higher SOD activity (Figure 7A). During the evaluation of POD and PPO activities, the POD and PPO activities were higher in the butanedione-treated group than in the blank control group, but lower in the treatment group inoculated with pathogenic fungus LB1. This means that PPO and POD activity in wolfberry fruit was kept to a minimum by 2,3-butanedione.

Through the preservation of fruits and vegetables, the activities of SOD, POD, and PPO are closely linked to physiological and biochemical processes (Xu et al., 2016). They are essential for fruit and vegetable stress resistance, postharvest color, and fineness preservation (Wang et al., 2004). The impacts of SOD enzyme activity revealed that the 2,3-butanedione remedy group had significantly higher SOD activity than the LB1 inoculation group. This is agreeing with the research outcomes of Chen (2018). 2,3-butanedione can delay senescence by inducing stress resistance in wolfberry fruits. POD and PPO play a vital function in oxidative metabolism in plants. PPO can oxidize phenolic compounds into quinone compounds that can stymie outgrowth of pathogenic microorganisms (Chen et al., 2019). 2,3-butanedione treatment group POD and PPO enzyme activities were lower than the pathogenic fungus LB1 group, but higher than the control group. 2,3-butanedione can effectively resist fungal attack, inhibit the growth and reproduction of the pathogenic strain LB1 in fruits and prolong the storage period of wolfberry.

## 4. Conclusion

Four strains of wolfberry pathogenic fungus were isolated, and 2,3-butanedione was confirmed to have antifungal activity and can suppress pathogenic fungi growth and reproduction in fresh wolfberry fruits after harvest, thus extending the storage time of fresh wolfberry fruit. The development of 2,3-butanedione as a post-harvest preservation method for wolfberry fruits has the potential to be significant.

## Data availability statement

The original contributions presented in the study are included in the article/Supplementary material, further inquiries can be directed to the corresponding author.

## Author contributions

All authors contributed to the study conception and design. HL, YZ, CY, and LL performed the material preparation, data collection, and analysis. The first draft of the manuscript was written by HL. All authors commented on previous versions of the manuscript. All authors contributed to the article and approved the submitted version.

## Funding

This work was supported by Lanzhou Science and Technology Plan Project 2018–1-104; Special Fund Project for Guiding Science and Technology Innovation and Development in Gansu Province 2019ZX-05; and Gansu Province’s Higher Education Industry Support Plan 2020C-21.

## Conflict of interest

The authors declare that the research was conducted in the absence of any commercial or financial relationships that could be construed as a potential conflict of interest.

## Publisher’s note

All claims expressed in this article are solely those of the authors and do not necessarily represent those of their affiliated organizations, or those of the publisher, the editors and the reviewers. Any product that may be evaluated in this article, or claim that may be made by its manufacturer, is not guaranteed or endorsed by the publisher.

## Footnotes

^1^ http://www.ncbi.nlm.nih.gov/blast

## References

[ref1] Ambler K. Brauw A. D. Godlonton S. (2017). Measuring postharvest losses at the farm level in Malawi. Australian J. Agricultural & Resource Econ. 62, 139–160. doi: 10.1111/1467-8489.12237

[ref3] Brilli F. Loreto F. Baccelli I. (2019). Exploiting plant volatile organic compounds (VOCs) in agriculture to improve sustainable defense strategies and productivity of crops. Front. Plant Sci. 10:264. doi: 10.3389/fpls.2019.00264, PMID: 30941152 PMC6434774

[ref4] Cenobio-Galindo A. D. J. Ocampo-López J. Reyes-Munguía A. Carrillo-Inungaray M. L. Cawood M. Medina-Pérez G. (2019). Influence of bioactive compounds incorporated in a Nanoemulsion as coating on avocado fruits (Persea americana) during postharvest storage: antioxidant activity, physicochemical changes and structural evaluation. Antioxidants 8:500. doi: 10.3390/antiox8100500 31640249 PMC6826954

[ref5] Chan H. H. L. Lam H. I. Choi K. Y. Li S. Z. C. Lakshmanan Y. Yu W. Y. (2019). Delay of cone degeneration in retinitis pigmentosa using a 12-month treatment with Lycium barbarum supplement. J. Ethnopharmacol. 236, 336–344. doi: 10.1016/j.jep.2019.03.023, PMID: 30877066

[ref6] Chaouachi M. Marzouk T. Jallouli S. Elkahoui S. Gentzbittel L. Ben C. (2021). Activity assessment of tomato endophytic bacteria bioactive compounds for the postharvest biocontrol of Botrytis cinerea. Postharvest Biol. Technol. 172:111389. doi: 10.1016/j.postharvbio.2020.11138

[ref7] Chen W. (2018). Effect of potato glycoside alkaloid on induced disease resistance and fresh-keeping of fresh wolfberry fruits. China: Gansu agricultural university.

[ref8] Chen C. Cai N. Chen J. (2019). Clove essential oil as an alternative approach to control postharvest blue mold caused by Penicillium italicum in citrus fruit. Biomol. Ther. 9, 1–13. doi: 10.3390/biom9050197 PMC6572225 31117317

[ref9] Chen M. Lin H. Zhang S. Lin Y. Chen Y. Lin Y. (2015). Effects of adenosine triphosphate (ATP) treatment on postharvest physiology, quality and storage behavior of Longan fruit. Food Bioprocess Technol. 8, 971–982. doi: 10.1007/s11947-014-1462-z

[ref10] Cui X. Ma D. Liu X. Zhang Z. Li B. Xu Y. (2021). Magnolol inhibits gray mold on postharvest fruit by inducing autophagic activity of Botrytis cinerea. Postharvest Biol. Technol. 180:111596. doi: 10.1016/j.postharvbio.2021.111596

[ref11] Fan X. J. Zhang B. Yan H. Feng J. T. Ma Z. Q. Zhang X. (2019). Effect of lotus leaf extract incorporated composite coating on the postharvest quality of fresh goji (Lycium barbarum L.) fruit. Postharvest Biol. Technol. 148, 132–140. doi: 10.1016/j.postharvbio.2018.10.020

[ref12] Farsalinos K. E. Kistler K. A. Gillman G. Voudris V. (2014). Evaluation of electronic cigarette liquids and aerosol for the presence of selected inhalation toxins. Nicotine Tob. Res. 17, 168–174. doi: 10.1093/ntr/ntu176, PMID: 25180080 PMC4892705

[ref13] Habibi F. Ramezanian A. Rahemi M. Eshghi S. Guillen F. Serrano M. (2019). Postharvest treatments with gamma-aminobutyric acid, methyl jasmonate, or methyl salicylate enhance chilling tolerance of blood orange fruit at prolonged cold storage. J. Sci. Food Agric. 99, 6408–6417. doi: 10.1002/jsfa.9920, PMID: 31283020

[ref14] Hallagan J. B. (2017). The use of diacetyl (2,3-butanedione) and related flavoring substances as flavorings added to foods-workplace safety issues. Toxicology 388, 1–6. doi: 10.1016/j.tox.2017.05.010, PMID: 28587783

[ref15] Jatoi M. A. Jurić S. Vidrih R. Vinceković M. Vuković M. Jemrić T. (2017). The effects of postharvest application of lecithin to improve storage potential and quality of fresh goji (Lycium barbarum L.) berries. Food Chem. 230, 241–249. doi: 10.1016/j.foodchem.2017.03.039, PMID: 28407907

[ref16] Kong J. Zhang Y. Ju J. Xie Y. Guo Y. Cheng Y. (2019). Antifungal effects of thymol and salicylic acid on cell membrane and mitochondria of Rhizopus stolonifer and their application in postharvest preservation of tomatoes. Food Chem. 285, 380–388. doi: 10.1016/j.foodchem.2019.01.099, PMID: 30797360

[ref17] Lan P. Gao F. R. Chen C. K. Wang W. S. Han J. Hai-Peng J. I. (2014). Separation and identification of pathogenic fungi from the postharvest Lycium Barbarmu. China Fruit & Vegetable 34, 9–12.

[ref18] Lemos Junior W. J. F. Binati R. L. Felis G. E. Slaghenaufi D. Ugliano M. Torriani S. (2020). Volatile organic compounds from Starmerella bacillaris to control gray mold on apples and modulate cider aroma profile. Food Microbiol. 89:103446. doi: 10.1016/j.fm.2020.103446, PMID: 32138994

[ref19] Li L. Hui P. Chen M. Zhang S. Zhong C. (2017). Isolation and identification of pathogenic fungi causing postharvest fruit rot of kiwifruit (Actinidia chinensis) in China. J. Phytopathol. 165, 782–790. doi: 10.1111/jph.12618

[ref20] Li Z. Wei Y. Xu Y. Han P. Jiang S. Xu F. (2021). Terpinen-4-ol treatment maintains quality of strawberry fruit during storage by regulating sucrose-induced anthocyanin accumulation. Postharvest Biol. Technol. 174:111461. doi: 10.1016/j.postharvbio.2020.111461

[ref21] Ling L. J. Li Z. B. Jiao Z. L. Zhang X. Ma W. X. Feng J. J. (2019). Identification of novel endophytic yeast strains from tangerine Peel. Curr. Microbiol. 76, 1066–1072. doi: 10.1007/s00284-019-01721-9, PMID: 31243536

[ref22] Ling L. Zhao Y. Tu Y. Yang C. Ma W. Feng S. (2021). The inhibitory effect of volatile organic compounds produced by Bacillus subtilis CL2 on pathogenic fungi of wolfberry. J. Basic Microbiol. 61, 110–121. doi: 10.1002/jobm.202000522, PMID: 33368461

[ref23] Liu W. J. Jiang H. F. Rehman F. U. Zhang J. W. Chang Y. Jing L. (2017a). Lycium Barbarum polysaccharides decrease hyperglycemia-aggravated ischemic brain injury through maintaining mitochondrial fission and fusion balance. Int. J. Biol. Sci. 13, 901–910. doi: 10.7150/ijbs.18404, PMID: 28808422 PMC5555107

[ref24] Liu J. Sui Y. Wisniewski M. Xie Z. G. Liu Y. Q. You Y. M. (2018). The impact of the postharvest environment on the viability and virulence of decay fungi. Crit. Rev. Food Sci. Nutr. 58, 1681–1687. doi: 10.1080/10408398.2017.1279122, PMID: 28140651

[ref25] Liu Y. Wang H. Wang Y. D. Sun W. Y. Guo X. X. Ran G. W. (2017b). Isolation, identification and biological characteristics of pathogenic fungus from Chinese wolfberry fruit. T. Chinese Society of Agricultural Engineering 33, 374–380.

[ref26] Liu J. Zhao Y. Xu H. Zhao X. Tan Y. Li P. (2021). Fruit softening correlates with enzymatic activities and compositional changes in fruit cell wall during growing in Lycium barbarum L. Int. J. Food Sci. Technol. 56, 3044–3054. doi: 10.1111/ijfs.14948

[ref27] Mari M. Bautista-Baños S. Sivakumar D. (2016). Decay control in the postharvest system: role of microbial and plant volatile organic compounds. Postharvest Biol. Technol. 122, 70–81. doi: 10.1016/j.postharvbio.2016.04.014

[ref28] María G. S. D. Gilda Y. A. M. Hugo C. A. B. Adrien G. (2018). Two efficient methods for isolation of high-quality genomic DNA from entomopathogenic fungi. J. Microbiol. Methods 148, 55–63. doi: 10.1016/j.mimet.2018.03.012 29596959

[ref29] Moore G. G. Lebar M. D. Carter-Wientjes C. H. Gilbert M. K. (2021). The potential role of fungal volatile organic compounds in aspergillus flavus biocontrol efficacy. Biol. Control 160:104686. doi: 10.1016/j.biocontrol.2021.104686

[ref30] Morgan D. L. Jokinen M. P. Johnson C. L. Price H. C. Gwinn W. M. Bousquet R. W. (2016). Chemical reactivity and respiratory toxicity of the alpha-Diketone flavoring agents: 2,3-Butanedione, 2,3-Pentanedione, and 2,3-Hexanedione. Toxicol. Pathol. 44, 763–783. doi: 10.1177/0192623316638962, PMID: 27025954 PMC5286456

[ref31] Nunes C. A. (2012). Biological control of postharvest diseases of fruit. Eur. J. Plant Pathol. 133, 181–196. doi: 10.1007/s10658-011-9919-7

[ref32] Sun G. Y. Cui J. Q. Wang S. F. Zhang R. Gleason M. L. (2008). First report of anthracnose of Lycium barbarum caused by Colletotrichum acutatum in China. Plant Dis. 92:1471. doi: 10.1094/PDIS-92-10-1471A, PMID: 30769541

[ref33] Tian J. Ban X. Zeng H. He J. Chen Y. Wang Y. (2012). The mechanism of antifungal action of essential oil from dill (Anethum graveolens L.) on Aspergillusflavus. PLoS One 7:e30147. doi: 10.1371/journal.pone.0030147, PMID: 22272289 PMC3260232

[ref34] Wallace R. L. Hirkala D. L. Nelson L. M. (2017). Postharvest biological control of blue mold of apple by Pseudomonas fluorescens during commercial storage and potential modes of action. Postharvest Biol. Technol. 133, 1–11. doi: 10.1016/j.postharvbio.2017.07.003

[ref35] Wang R. Q. Feng J. H. Wei W. W. Xu X. M. Qing Y. W. (2012). Effect of 1-methylcyclopropene and modified atmosphere packaging on quality retention during cold-tempereture storage of Lycium barbarum fruit. Transactions of the Chinese Society of Agricultural Engineering 28, 287–292.

[ref36] Wang Y. S. Tian S. P. Xu Y. Qin G. Z. Yao H. (2004). Changes in the activities of pro-and anti-oxidant enzymes in peach fruit inoculated with Cryptococcus laurentii or Penicillium expansum at 0 or 20 °C. Postharvest Biol. Technol. 34, 21–28. doi: 10.1016/j.postharvbio.2004.04.003

[ref37] Wang Y. M. Zhang K. Fei-Hua X. U. Wang Y. Ren X. W. Zhang B. L. (2014). Chemical analysis and nutritional evaluation of different varieties of goji berries (Lycium barbarum L.). Food Sci. 35, 34–38.

[ref38] Wang Z. Zhong T. Chen K. Du M. Chen G. Chen X. (2020). Antifungal activity of volatile organic compounds produced by Pseudomonas fluorescens ZX and potential biocontrol of blue mold decay on postharvest citrus. Food Control 107499:310. doi: 10.1016/j.foodcont.2020.107499

[ref39] Wei L. Mao W. Jia M. Xing S. Ali U. Zhao Y. (2018). FaMYB44.2, a transcriptional repressor, negatively regulates sucrose accumulation in strawberry receptacles through interplay with FaMYB10. J. Exp. Bot. 69, 4805–4820. doi: 10.1093/jxb/ery249, PMID: 30085079 PMC6137983

[ref40] Wonglom P. Ito S. Sunpapao A. (2020). Volatile organic compounds emitted from endophytic fungus Trichoderma asperellum T1 mediate antifungal activity, defense response and promote plant growth in lettuce (Lactuca sativa). Fungal Ecol. 43:100867. doi: 10.1016/j.funeco.2019.100867

[ref41] Wu Q. Gao H. Zhang Z. Li T. Qu H. Jiang Y. (2020). Deciphering the metabolic pathways of pitaya Peel after postharvest red light irradiation. Meta 10:108. doi: 10.3390/metabo10030108, PMID: 32183356 PMC7143668

[ref42] Xiao W. Di X. X. Yong C. Ling Z. R. Ping T. S. Qiang L. B. (2018). Isolation and identification of postharvest pathengens in fresh wolfberry from Ningxia and inhibitory effect of salicylic acid. J. Food Safety and Quaity 9, 5837–5842.

[ref43] Xu F. Wang S. Xu J. Liu S. Li G. (2016). Effects of combined aqueous chlorine dioxide and UV-C on shelf-life quality of blueberries. Postharvest Biol. Technol. 117, 125–131. doi: 10.1016/j.postharvbio.2016.01.012

[ref410] Xu Y. Wei J. Wei Y. Han P. Dai K. Zou X. (2021). Tea tree oil controls brown rot in peaches by damaging the cell membrane of Monilinia fructicola. Postharvest Biol. Technol. 175:111474. doi: 10.1016/j.postharvbio.2021.111474, PMID: 32183356

[ref44] Yang S. Hong W. C. Jie C. Z. Ting H. Y. (2016). Research Progress on postharvest physiology and storage Technology of Fresh Fruit of Lycium barbarum L. Storage and Process 16, 102–106.

[ref45] Zhang S. W. Zheng Q. Xu B. L. Liu J. (2019). Identification of the fungal pathogens of postharvest disease on peach fruits and the control mechanisms of Bacillus subtilis JK-14. Toxins 11:322. doi: 10.3390/toxins11060322, PMID: 31195675 PMC6628418

[ref46] Zhao P. Y. Li P. Z. Wu S. Y. Zhou M. S. Gao H. Y. (2019). Volatile organic compounds (VOCs) from Bacillus subtilis CF-3 reduce anthracnose and elicit active defense responses in harvested litchi fruits. AMB Express 9:119. doi: 10.1186/s13568-019-0841-2, PMID: 31352537 PMC6661035

[ref47] Zhong T. Wang Z. Zhang M. Wei X. Kan J. Zalán Z. (2021). Volatile organic compounds produced by Pseudomonas fluorescens ZX as potential biological fumigants against gray mold on postharvest grapes. Biol. Control 163:104754. doi: 10.1016/j.biocontrol.2021.104754

[ref48] Zhou D. Wang Z. Li M. Xing M. Xian T. Tu K. (2017). Carvacrol and eugenol effectively inhibit Rhizopus stolonifer and control postharvest soft rot decay in peaches. J. Appl. Microbiol. 124, 166–178. doi: 10.1111/jam.13612, PMID: 29044849

