# Supplementary material for: Fungal pathogens causing postharvest fruit rot of wolfberry and inhibitory effect of 2,3-butanedione
Source: Front Microbiol. 2023 Jan 10;13:1068144. doi: 10.3389/fmicb.2022.1068144 (PMC9871540; doi:10.3389/fmicb.2022.1068144)
Supplement: Supplementary file 1 [file Table_1.docx]

Table 1 Determine the amount of SOD active reagent added

| reagent (enzyme) | | amount/mL | reagent (enzyme) | amount/mL |
| --- | --- | --- | --- | --- |
| 50 m mol/L、pH 7.8 phosphate buffer | 1.7 | | 100 μmol/L EDTA-Na_2_ | 0.3 |
| 130 m mol/L、MET solution | 0.3 | | 100 μmol/L riboflavin solution | 0.3 |
| 750 μmol/L NBT olution | 0.3 | | enzyme solution | 0.1 |
